# Supplementary material for: The human Toll-like receptor 2 (TLR2) response during pathogenic Leptospira infection
Source: bioRxiv. 2023 Nov 17:2023.11.16.567338. Preprint. [Version 1] doi: 10.1101/2023.11.16.567338 (PMC10680769; doi:10.1101/2023.11.16.567338)
Supplement: 1 [file NIHPP2023.11.16.567338v1-supplement-1.pdf]

bioRxiv preprint doi: https://doi.org/10.1101/2023.11.16.567338; this version posted November 17, 2023. The copyright holder for this preprint (which was not certified by peer review) is the author/funder, who has granted bioRxiv a license to display the preprint in perpetuity. It is made available under aCC-BY 4.0 International license.

Supporting information

S1 Appendix. Primer Designing steps-Human TLR2 primer pair 2

S2 Appendix. Primer Designing steps-Human TLR2 primer pair 1

S3 Appendix. Primer Designing steps-Human HPRT1

S4 Appendix. Primer Designing steps-Human B2M

S1 Figure. Visualized PCR bands of human TLR2 primer products on a 2% Agarose gel containing Ethidium bromide

bioRxiv preprint doi: https://doi.org/10.1101/2023.11.16.567338; this version posted November 17, 2023. The copyright holder for this preprint (which was not certified by peer review) is the author/funder, who has granted bioRxiv a license to display the preprint in perpetuity. It is made available under aCC-BY 4.0 International license.

S2 Figure. Visualized PCR bands on a 2% Agarose gel containing Ethidium Bromide

S3 Figure. Relative normalized expression of TLR2 amplified using Primer pair I (right hand side) and Primer pair II (left hand side)

S4 Figure. The Relative normalized expression of human TLR2 gene (PO47 showed expression)

S5 Figure. The Relative normalized expression of human TLR2 gene (PO47 showed expression)

S6 Figure. The Relative normalized expression of human TLR2 gene (reference B2M)S1,S6, S8

S7 Figure. The Relative normalized expression of human TLR2 gene (referenceHPRT1) S1,S6,S8
